# The Silence of PSMC6 Inhibits Cell Growth and Metastasis in Lung Adenocarcinoma

**DOI:** 10.1155/2021/9922185

**Published:** 2021-06-19

**Authors:** Jian-Yu Zhang, Ke-Zhi Shi, Xiang-Yu Liao, Shi-Jun Li, Dan Bao, Ying Qian, Dao-Jun Li

**Affiliations:** The First College of Clinical Medical Science, Three Gorges University & Yichang Central People's Hospital, Yichang, China

## Abstract

The proteasome has been validated as an anticancer drug target, while the role of a subunit of proteasome, PSMC6, in lung adenocarcinoma (LUAD) has not been fully unveiled. In this study, we observed that both the RNA and protein of PSMC6 were highly upregulated in LUAD compared with the adjacent normal tissues. Moreover, a high PSMC6 expression was associated with poor prognosis. In accordance with this finding, PSMC6 was associated with poor tumor differentiation. Furthermore, the silence of PSMC6 by small interference RNAs (siRNAs) could significantly inhibit cell growth, migration, and invasion in lung cancer cell lines, suggesting that PSMC6 might serve as a promising therapeutic target in LUAD. To further explore the molecular mechanism of PSMC6 in LUAD, we observed that the proteasome subunits, such as PSMD10, PSMD6, PSMD9, PSMD13, PSMB3, PSMB1, PSMA4, PSMC1, PSMC2, PSMD7, and PSMD14, were highly correlated with PSMC6 expression. Based on the gene set enrichment analysis, we observed that these proteasome subunits were involved in the degradation of AXIN protein. The correlation analysis revealed that the positively correlated genes with PSMC6 were highly enriched in WNT signaling-related pathways, demonstrating that the PSMC6 overexpression may activate WNT signaling via degrading the AXIN protein, thereby promoting tumor progression. In summary, we systematically evaluated the differential expression levels and prognostic values of PSMC6 and predicted its biological function in LUAD, which suggested that PSMC6 might act as a promising therapeutic target in LUAD.

## 1. Introduction

Lung cancer is among the most frequent malignancies worldwide, accounting for nearly 20% of cancer-related deaths in 2018 [1]. The major risk factors for lung cancer are smoking, radon exposure, and exposure to other carcinogens [2, 3]. Patients with lung cancer often had unfavorable outcomes, and the 5-year survival rate for lung cancer remains less than 20% [4]. Novel treatments and drug designs are direly sought to improve patients' prognoses and relieve their financial burden [5].

Proteasome inhibition is considered a promising treatment strategy for various malignancies, including lung cancer. The 26S proteasome is an important protease in eukaryotic cells, which is composed of a 20S core particle (CP) and one or two 19S regulatory particles (RP) capping one or both ends of the 20S CP [6]. The 26S proteasome mediates degradation of numerous cellular proteins and participates in multiple cellular processes, especially the cell cycle [7], which makes it a potential target in cancer therapy.

26S proteasome assembly induced by PSMD5 inactivation is observed during colorectal tumor progression, and it has been further validated that reduced 26S proteasome levels could impair cancer cell viability and that partial depletion of the 19S RP subunits could effectively result in inhibition of the 26S proteasome [8, 9]. Those subunits are essential for the 19S RP to carry out functions such as identification, binding, deubiquitination, unfolding, and translocation of substrates before proteolysis [10, 11]. Receptor RPN13 in the 19S regulatory particle is found overexpressed in ovarian and colon cancer, and it could interact with RA190, a bis-benzylidine piperidone active against cervical and ovarian cancer [12–14]. Of note, PSMC6 codes for one of the six AAA-type ATPase subunits of the 19S RP and has been identified as a protective gene in lower grade glioma [15, 16]. Its high bortezomib sensitivity makes it the most prominent target in multiple myeloma [17]. A recent study has demonstrated that PSMC6 overexpression could impair cell cycle progression and cell proliferation via inhibiting the PI3K/AKT signaling pathway [18]. Meanwhile, S5aC, a multiubiquitin binding component of the 19S RP, is found capable of inducing A549 lung cancer cell death [19]. Therefore, a closer investigation of genes related to the 26S proteasome in lung cancer shall provide detailed information on the cellular functions of those subunits in lung cancer carcinogenesis and reveal potential therapeutic targets. In the present study, we investigated the clinical and functional relevance of PSMC6 in lung adenocarcinoma (LUAD) and explored its underlying mechanism in the initiation and progression of LUAD.

## 2. Materials and Methods

### 2.1. Data Acquisition

The gene expression data of The Cancer Genome Atlas (TCGA) and the protein expression data were downloaded from the UCSC Xena database [20] and an earlier study [21]. The Fragment Per Kilo-Million (FPKM) and read count-based data were collected from the TCGA cohort. Briefly, the raw fastq data were aligned to reference genome by STAR v2 [22] and gene expression levels were quantified by HTSeq [23]. The gene-level protein intensities were collected, imputed by the minimal protein intensity, and logarithm transformed.

### 2.2. Differential Expression

The differential expression between two groups was conducted by Wilcoxon rank sum test, while the multisample comparison was tested by the Kruskal-Wallis test. Moreover, the fold change was also employed to test the difference.

### 2.3. Survival Analysis

The Cox proportional hazard regression model was used to evaluate the association between PSMC6 expression and survival time. Particularly, the PSMC6 expression was discretized as high and low expression levels using the median as cut-off. The survival analysis was implemented in R survival package (https://cran.r-project.org/web/packages/survival/index.html).

### 2.4. Functional Inference of PSMC6

The prediction of the biological function for PSMC6 was conducted by integrating the correlation analysis, protein-protein interaction (PPI) analysis, and gene set enrichment analysis (GSEA). The PPI data was obtained from the BioGRID database [24]. Specifically, we first extracted the proteins (genes) directly interacting with PSMC6 from the PPI network. Secondly, those interacting proteins that showed a significantly positive correlation with PSMC6 were retained for the next step analysis (*p* value < 0.05, Spearman correlation > 0.3). Thirdly, those genes were subjected to the gene set enrichment analysis (GSEA) against the pathways curated from the Reactome database, and hypergeometric test was employed to test the statistical significance of the GSEA. The GSEA was implemented in the R clusterProfiler package [25].

### 2.5. Cell Culture, RNA Isolation, and Quantitative Real-Time PCR (qRT-PCR)

The cells were cultured following a previous study [26]. Total RNA was isolated from the A549 and H1299 cell lines using TRIzol reagent (Sangon, China). The reverse transcription of the RNAs was performed to synthesize the cDNAs following the instructions of PrimeScript™ RT reagent Kit (Takara Bio Inc.). The mRNA expression was quantified by qRT-PCR using SYBR premix Ex Taq II with LightCycler 480II (Roche) instrument. The sequences of the primers are as follows: PSMC6 forward, 5-CGGGTGAAAGTGCTCGTTTG-3 and reverse, 5-AGCAAAGCAGGATCCAGTGT-3 and GAPDH forward, 5-GTCGTGGAGTCTACTGGTGTC-3 and reverse, 5-GAGCCCTTCCACAATGCCAAA-3. All these experiments were conducted in triplicates.

### 2.6. RNA Interference and Transfection

We purchased the synthetic PSMC6 siRNAs and its negative control (NC) at the concentration of 100 nM from GenePharma (Shanghai, China). Specific siRNAs targeting PSMC6 are as foloows: si-PSMC6 #1: 5′-ACAAGGAGATCGACGGCCGTCTTAA-3′ and si-PSMC6 #2: 5′-CGGCCGTCTTAAGGAGTTAAGGGAA-3′. Following the manufacturer's procedure, the transfection was conducted with Lipofectamine 2000 Transfection Reagent (Life, USA), which was purchased from Life Technologies. All these experiments were conducted in triplicates.

### 2.7. Cell Counting Kit-8 (CCK-8) Analysis

The CCK-8 assay was used to determine the cell proliferation level following the method from a previous study [26]. All these experiments were conducted in triplicates.

### 2.8. Cell Invasion and Migration Assays

The cell invasion and migration assays were performed following the method of a previous study [27]. Specifically, Transwell plates (8 *μ*m pore size, 6.5 mm diameter; Corning, USA) precoated with Matrigel Basement Membrane Matrix (coating concentration: 1 mg/ml; BD Biosciences, Franklin Lakes, NJ) were used for the migration assay according to the manufacturer's protocol. Subsequently, the media containing 1% FBS into the upper chamber of the Transwell filter on a 24-well plate were used for cell seeding after transfection, and those containing 10% FBS into the lower well of the plate were used as an attractant. After 72 h of incubation, cells on the upper side of the filters or migrated to the lower side were removed or fixed with methanol, stained with Giemsa, and counted under a microscope. Migration assays were performed with the same procedure, except that the Transwell chamber inserts were not coated with Matrigel, and the medium containing 10% FBS was used for cell suspensions. All these experiments were conducted in triplicates.

## 3. Results

### 3.1. PSMC6 Is a Poor Prognosis in Lung Adenocarcinoma

**Figure 1:**
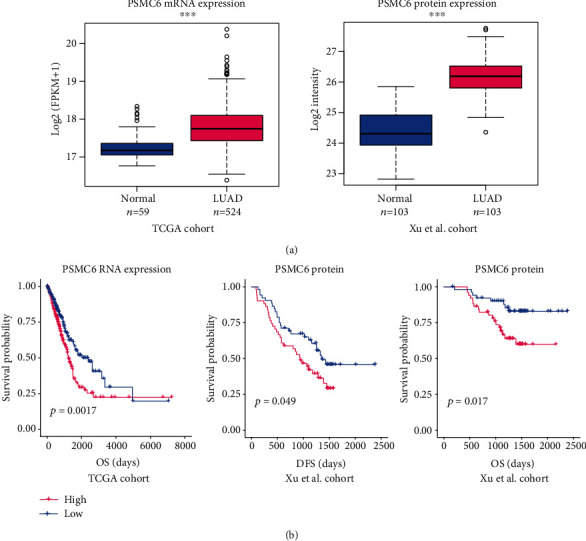
The upregulation of PSMC6 in lung adenocarcinoma (LUAD). (a) The RNA and protein expression levels of PSMC6 in LUAD and adjacent normal tissues. (b) The correlation between survival time and PSMC6 RNA or protein expression levels. The red and blue lines indicate the samples with high and low PSMC6 expression. DFS: disease-free survival; OS: overall survival.

To reveal the expression pattern of PSMC6 in lung adenocarcinoma (LUAD), we collected two cohorts from the Cancer Genome Atlas (TCGA) and Xu et al. and evaluated its differential expression levels between the tumor and normal tissues. Specifically, the mRNA and protein expressions of PSMC6 were highly upregulated in LUAD as compared with the adjacent normal tissues (Figure 1(a), Wilcoxon rank sum test, *p* value < 0.001). Notably, the PSMC6 protein was expressed over fourfold in LUAD than that in the normal tissues (Figure 1(a)).

Moreover, the tumor samples were stratified into the high- and low-expression groups. The survival analysis of PSMC6 RNA and protein expressions revealed that patients with high PSMC6 RNA expression had shorter overall survival (OS) than those with a low expression (Figure 1(b), log-rank test, *p* value < 0.05). Consistently, the LUAD samples with high PSMC6 protein expression had both shorter disease-free survival (DFS) and OS than those with low PSMC6 protein expression (Figure 1(b), log-rank test, *p* value < 0.05). These results indicated that PSMC6 overexpression might result in a worse prognosis and act as a prognostic biomarker in LUAD.

### 3.2. The Association of PSMC6 with the Clinical Characteristics

**Figure 2:**
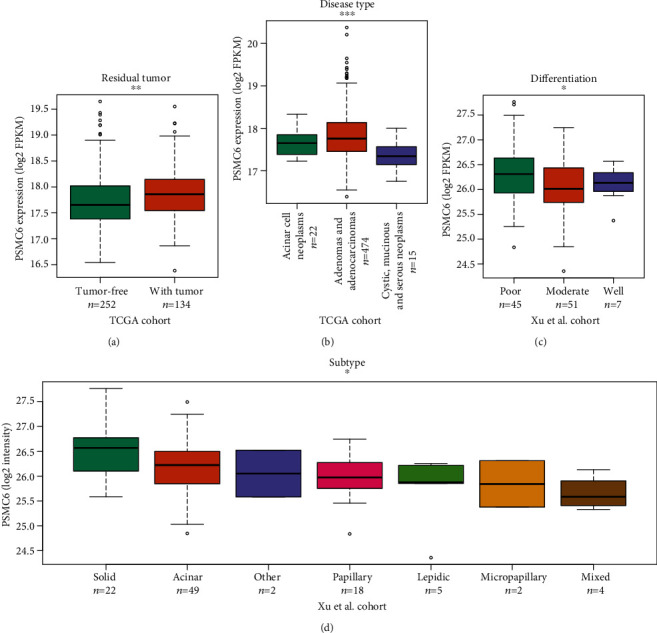
The association of PSMC6 expression with clinical factors. The association of PSMC6 expression with residual tumor, disease type, differentiation, and subtype are displayed in (a), (b), (c), and (d), respectively. ^∗^, ^∗∗^, and ^∗∗∗^ represent the *p* values below 0.05, 0.01, and 0.001.

To evaluate the clinical significance of PSMC6 in LUAD, we compared the RNA or protein expression of PSMC6 of tumor samples with different clinical characteristics. Notably, PSMC6 was expressed higher in LUAD with residual tumor than those without (Figure 2(a), *p* value < 0.01), suggesting that PSMC6 was associated with residual tumor, which was considered a risk factor of tumor recurrence [28, 29]. Among the three disease types, LUAD with adenomas and adenocarcinomas had a higher RNA expression of PSMC6 than the other two disease types (Figure 2(b), Kruskal-Wallis test, *p* value < 0.001), indicating that LUAD with the disease type of adenomas and adenocarcinomas might have a higher degree of malignancy. Consistently, PSMC6 RNA expression was higher in the LUAD patients with poorly differentiated tumor than those with well and moderately differentiated tumors (Figure 2(c)), suggesting that PSMC6 was associated with the tumor differentiation. Furthermore, among the 7 major subtypes of LUAD, solid adenocarcinoma of the lung had the highest protein expression of PSMC6 (Figure 2(d)), suggesting that solid adenocarcinoma of the lung might have a relatively worse prognosis than other subtypes. These results disclosed that PSMC6 was clinically relevant to factors including residual tumor, disease type, tumor differentiation, and LUAD subtype.

### 3.3. Silence of PSMC6 Inhibits Cell Proliferation of Non-Small-Cell Lung Cancers

**Figure 3:**
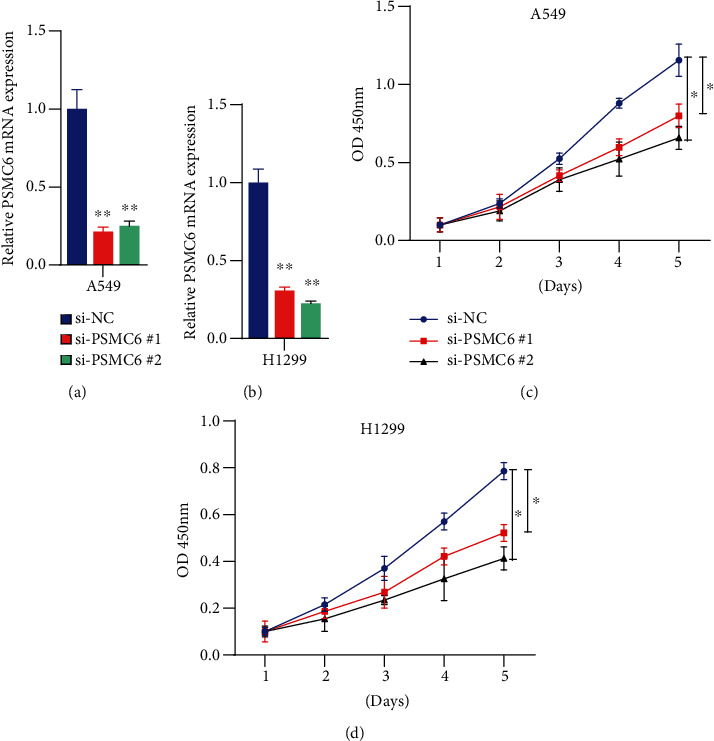
The impact of PSMC6 silence on the tumor cell proliferation. The relative mRNA expression of PSMC6 in negative controls and cell lines with siRNA treatments (si-PSMC6 #1 or #2). The experiments for A549 and H1299 cell lines are shown in (a) and (b), respectively. The cell proliferation levels of A549 and H1299 with and without siRNA treatments (si-PSMC6 #1 and si-PSMC6 #2) are displayed in (c) and (d). ^∗^, ^∗∗^, and ^∗∗∗^ represent the *p* values below 0.05, 0.01, and 0.001.

To uncover the functional role of PSMC6 in non-small-cell lung cancer (NSCLC), we performed CCK-8 assay to test the impact of PSMC6 on the cell proliferation (Materials and Methods). Specifically, we designed two small interface RNAs (siRNA) for PSMC6 mRNA, termed as si-PSMC6 #1 and si-PSMC6 #2, and transfected into two NSCLC cell lines, A549 and H1299. As shown in Figures 3(a) and 3(b), the siRNA transfection could efficiently suppress the RNA expression levels of PSMC6 in the two cell lines (*p* value < 0.01) using quantitative real-time polymerase chain reaction (qPCR). With the siRNA transfection, the cell proliferation levels were found to be significantly inhibited at the fifth day (Figures 3(c) and 3(d), *p* value < 0.05). These results demonstrated that silence of PSMC6 could efficiently inhibit the cell proliferation of NSCLC.

### 3.4. Silence of PSMC6 Inhibits Migratory and Invasive Abilities of Non-Small-Cell Lung Cancer Cells

**Figure 4:**
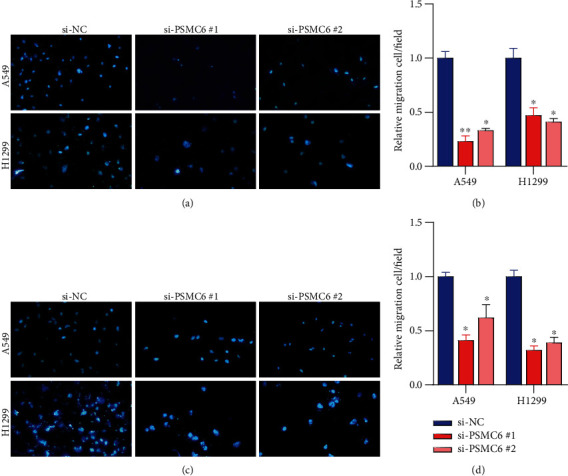
The impact of PSMC6 silence on cell invasion and migration. The cell invasion (a, b) and migration (c, d) of A549 and H1299 after negative controls and siRNA transfections are counted by Transwell assay. ^∗^, ^∗∗^, and ^∗∗∗^ represent the *p* values below 0.05, 0.01, and 0.001.

As PSMC6 was negatively associated with survival time of LUAD patients, we then investigated whether silence of PSMC6 could restrict the migratory and invasive abilities of NSCLC cells. Expectedly, the number of migratory cancer cells was obviously decreased in the cells with si-PSMC6 transfection than the negative controls (Figure 4(a)). The quantitative analysis revealed that the number of migratory cells with si-PSMC6 transfection was greater in the cells with si-PSMC6 treatment (Figure 4(b)). Consistently, the tumor cell invasion was also inhibited by the PSMC6 silence (Figures 4(c) and 4(d)). These results indicated that silence of PSMC6 could inhibit migratory and invasive abilities of cancer cells.

### 3.5. The Proteasome Might Activate WNT Signaling via Degrading AXIN Protein

**Figure 5:**
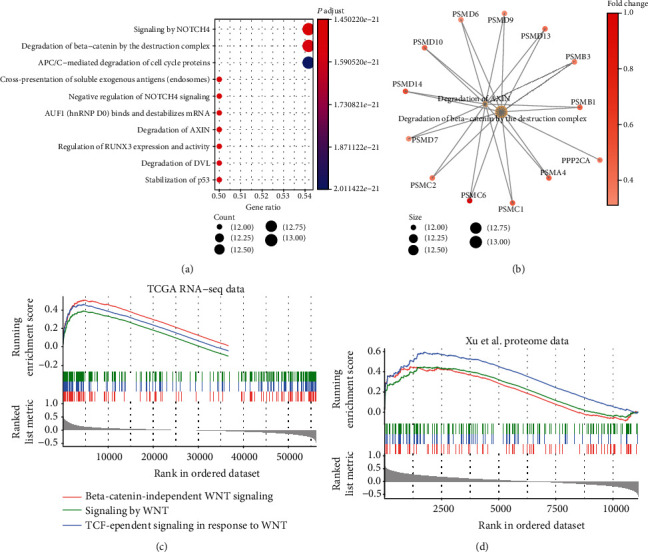
The proteins interacting with PSMC6 and the downstream pathways. (a) The pathway enriched by the proteins interacting with PSMC6. The node color and size represent the *p* value and number of genes within the pathways. (b) The WNT signaling-related pathways enriched by the proteasome subunits interacting with PSMC6. (c, d) The correlation between the PSMC6 and the genes involved in WNT signaling pathways. The lines under the curves represent the genes involved in the WNT signaling pathways, which were ranked by their correlation coefficients with PSMC6. The coefficients are represented by the grey bars.

To gain insights into the molecular mechanism of PSMC6 in LUAD, we conducted a correlation analysis between PSMC6 and other genes using both RNA-seq and proteome data. Totally, we identified 1222 genes coexpressed with PSMC6 (Spearman correlation > 0.3), of which, 26 genes encoded proteins interacting with PSMC6 protein. The gene set enrichment analysis revealed that degradation of beta-catenin by the destruction complex and degradation of AXIN was significantly enriched by these 26 genes (Figure 5(a)). Specifically, the proteasome subunits such as PSMD10, PSMD6, PSMD9, PSMD13, PSMB3, PSMB1, PSMA4, PSMC1, PSMC2, PSMD7, and PSMD14 were involved in those two pathways (Figure 5(b)). As the AXIN protein acted as a tumor suppressor to inhibit WNT signaling pathway, its degradation might result in WNT signaling activation. Consistently, the positively correlated genes with PSMC6 were highly enriched in WNT signaling-related pathways such as beta-catenin-independent WNT signaling, signaling by WNT, and TCF-dependent signaling in response to WNT at both RNA (Figure 5(c), FDR < 0.05) and protein (Figure 5(d), FDR < 0.05) levels. These results indicated that PSMC6 might activate WNT signaling via degrading AXIN protein.

## 4. Discussion

The proteasome has been validated as an anticancer drug target [30], while the role of a subunit of proteasome, PSMC6, in lung adenocarcinoma (LUAD) has not been fully unveiled. In this study, we observed that both the RNA and protein of PSMC6 were highly upregulated in LUAD compared with the adjacent normal tissues. Moreover, high PSMC6 expression was associated with poor prognosis. To our knowledge, previous studies rarely reported this finding. However, the other subunits of proteasome, such as PSMD3, PSMC2, and PSMD4, were upregulated in several cancers and associated with prognosis [31–33]. After systematic treatments, some LUAD patients might still have residual tumors, which had been considered a risk factor of recurrence [28, 29]. In accordance with this finding, PSMC6 was associated with poor tumor differentiation, suggesting that high expression of PSMC6 in patients with residual tumors or poor tumor differentiation indicates that PSMC6 may be associated with tumor recurrence. Moreover, the PSMC6 was also observed to have higher expression levels in some histology subtypes such as adenomas/adenocarcinomas and solid tumor subtypes. The solid predominant subtype of LUAD has been observed to have much worse prognosis than other subtypes [34].

Moreover, the silence of PSMC6 by siRNA could significantly inhibit cell growth, migration, and invasion in lung cancer cell lines. Consistently, PSMC6 was also identified as a target for bortezomib sensitivity in multiple myeloma by CRISPR genome-wide screening [16]. We thus speculated that PSMC6 might serve as a promising therapeutic target in LUAD.

To further explore the molecular mechanism of PSMC6 in LUAD, we observed that the proteasome subunits, such as PSMD10, PSMD6, PSMD9, PSMD13, PSMB3, PSMB1, PSMA4, PSMC1, PSMC2, PSMD7, and PSMD14, were highly correlated with PSMC6 expression. It should be noted that these proteins could directly interact with PSMC6 and act as components of proteasome. Among these proteasome subunits, PSMB3 [35] and PSMD14 [36] have been found to promote lung adenocarcinoma progression, while PSMA4 polymorphisms are associated with lung cancer susceptibility and response to cisplatin-based chemotherapy [37], suggesting that the proteasome may be associated with the LUAD progression and drug response due to numerous subunits. Based on the gene set enrichment analysis, we observed that these proteasome subunits were involved in the degradation of the AXIN protein. The correlation analysis revealed that the positively correlated genes with PSMC6 were highly enriched in WNT signaling-related pathways. The activity of WNT signaling was enhanced by the degradation of the AXIN complex via the proteasome [38], further demonstrating that the PSMC6 overexpression may activate WNT signaling via degrading AXIN protein, thereby promoting tumor progression. However, this mechanism needs to be validated by more experimental data.

In summary, we systematically evaluated the differential expression levels and prognostic values of PSMC6 and predicted its biological function in LUAD, which suggested that PSMC6 might act as a promising therapeutic target in LUAD.

## Data Availability

All data supporting this study are collected from a public database such as TCGA and Gene Expression Omnibus (GEO), which have been cited as references in Materials and Methods.
